# Exploiting and integrating rich features for biological literature classification

**DOI:** 10.1186/1471-2105-9-S3-S4

**Published:** 2008-04-11

**Authors:** Hongning Wang, Minlie Huang, Shilin Ding, Xiaoyan Zhu

**Affiliations:** 1 State Key Laboratory of Intelligent Technology and Systems Tsinghua National Laboratory for Information Science and Technology Department of Computer Science and Technology, Tsinghua University, Beijing 100084, China

## Abstract

**Background:**

Efficient features play an important role in automated text classification, which definitely facilitates the access of large-scale data. In the bioscience field, biological structures and terminologies are described by a large number of features; domain dependent features would significantly improve the classification performance. How to effectively select and integrate different types of features to improve the biological literature classification performance is the major issue studied in this paper.

**Results:**

To efficiently classify the biological literatures, we propose a novel feature value schema TF*ML, features covering from lower level domain independent “string feature” to higher level domain dependent “semantic template feature”, and proper integrations among the features. Compared to our previous approaches, the performance is improved in terms of *AUC* and *F-Score* by 11.5% and 8.8% respectively, and outperforms the best performance achieved in BioCreAtIvE 2006.

**Conclusions:**

Different types of features possess different discriminative capabilities in literature classification; proper integration of domain independent and dependent features would significantly improve the performance and overcome the over-fitting on data distribution.

## Background

In the general text classification, effective feature is essential to make the learning task more efficient and accurate. No degree of classifiers can make up for a lack of predictive information in the input features [1]. In bioscientific literature, where biological structures and terminologies are described in a large number of features, the situation is more serious: well-chosen features could improve the classification accuracy substantially and decrease the risk of over-fitting [2].

In the early days of biological literature classification study, most of the researchers depended on the domain experts to pick out the informative features. Regev et al. used expert-defined rules to extract features from the semi-structure text and figure legends. Besides, they utilized external lexical resources and semantic constraints to achieve a better coverage and accuracy [3]. Min Shi et al. employed two types of keywords as feature: one type was from the given evidences and the other type was manually extracted from the training texts by domain experts [4]. Moustafa M. Ghanem et al. utilized expert-edited regular expressions to capture frequently occurring keyword combinations (or motifs) within short segments of the text in a document [5]. All these approaches require the involvement of domain experts in identifying the specific textual objects and the informative templates, so that they cannot easily be automatically extended to an efficient and scale-free model on other biological datasets [6].

Recent years, fully automatic and scalable text classification algorithm provides an alternative to the previous methods. Wilbur employed unigram, bigram and all of the *MeSH* terms as the set of feature to represent the documents [7]. Dobrokhotv et al. utilized the words processed by the XEROX natural language processing tool as discriminating attributes [8]. Aaron et al. used “Bag of Words” model: content was tokenized and stemmed into unigram feature and modelled the samples as binary feature vectors [9].

Although all of these features catch some aspects of biological and statistical meanings, they still cannot well and automatically exploit the domain dependent information from the complex biological literature. It becomes a challenge in biological text mining field to automatically introduce higher level domain dependent features into the classification process and integrate with the lower level domain independent features.

In this paper, we investigate the issue of biological literature classification from the perspective of feature selection and integration, which is evaluated by BioCreAtIvE [10], an international evaluation in biological text mining. In IAS (Protein Interaction Article Sub-task) of BioCreAtIvE 2006, participants were asked to classify a given set of *MEDLINE* titles and abstracts, according to whether a document contains at least one physical PPI (Protein Protein Interaction) or not. This procedure would be extremely useful for facilitating the efficiency of manual curation since it will largely filter out the irrelevant documents. In the evaluation, one of our implemented classifiers achieved outstanding results: the *Accuracy* ranked at the 1^st^ place, *AUC* and *F-Score* ranked at the 2^nd^ place respectively.

Although the result is encouraging, the performance has dropped significantly from the 5-fold cross validation on the training set to the evaluation on the official testing set (15.2% lower by *AUC*, 11.8% lower by *F-Score*). Main differences between these two data sets are: 1) the testing documents are mainly published in 2006 while the training documents distributes evenly over the past years; 2) the relevant/irrelevant document rate in the training set is nearly 2:1 while in the testing set it is 1:1. To statistically analyze the phenomenon, we use the variance of *Kullback Leibler* divergence to estimate the distribution of the top 50 employed features on the training and testing sets as follows:

$$KL`\left(P,Q\right)=\frac{1}{2}\sum_{x}\left(P\left(x\right)\log\frac{P\left(x\right)}{Q\left(x\right)}+Q\left(x\right)\log\frac{Q\left(x\right)}{P\left(x\right)}\right)$$

where *x* is the word and phrase features employed in IAS, *P(x)* and *Q(x)* are the probability of *x* in the training and testing set respectively.

**Figure 1:**
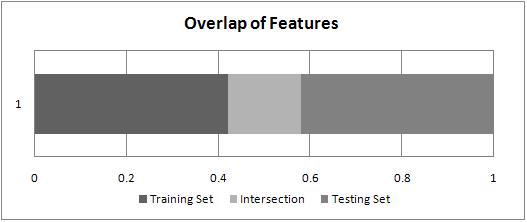
Overlap of Features between Training and Testing Set (Top 300 selected distinct features from the training and testing set according to *Chi-Square* statistics respectively)

The result (see Table 1) demonstrates that there is great divergence between the probability distribution of features in the irrelevant document set. And only one thirds of the top 300 features selected from the training set accordingly occur in the testing set (see Figure 1). It is clear that our previously selected features are limited and sensitive to the data distribution. How to efficiently exploit the domain independent and dependent features in the biological literature and avoid the over-dependence on data distribution motivates us to have an in-depth investigation in this paper.

**Table 1:** KL Divergence on Training, Cross Validation and Testing Set

***Unigram Feature***	***Relevant Probability***	***Irrelevant Probability***
*Training Set Vs Cross Validation Set*	0.0216	0.0703
*Training Set Vs Testing Set*	0.0369	**0.9926**

(Top 50 features according to *Chi-Square* statistics)

The rest of the paper is organized as follows. We will introduce the detailed description of methodologies proposed in this paper in Methods section. In Results and discussion section, we will present the experiment results and analysis. In Conclusion section, we will summarize our contributions in this paper.

## Methods

In this paper, we are engaged to investigate the issue from the perspective of feature selection and integration. The main contribution in this paper lies in that: we propose 1). domain independent feature value schema TF*ML and length-fixed string feature 2). domain dependent “semantic template” feature 3). efficient integrations among the features. These methods are described respectively in the following.

### Probabilistic schema

The traditional TF*IDF schema [11] just takes into consideration the occurrences of words in the whole corpus, while discarding the distribution of words in different categories. Differently, we propose a novel probabilistic feature value schema TF*ML (production of Term Frequency by Maximum Likelihood) to substitute the traditional TF*IDF as follows:

$$TF\;\left(t\right)\;\times \;ML\left(t\right)\;=\;TF\;\left(t\right)\;\times \;\left(\log\;P\left(t|c^{+}\right)\;-\;\log\;P\left(t|c^{-}\right)\right)$$

where *t* means the selected feature word, *c*^+^ and *c*^−^ mean the relevant and irrelevant category, *P(t|c^+^)* and *P(t|c^−^)* mean the probability that *t* occurs in category *c*^+^*andc*^−^ respectively.

The sign of *ML* indicates the category relevance of the feature and the magnitude reflects the classification confidence. Following the same idea as TF*IDF to express the specificities of features in different documents, we also multiply *TF* by *ML*.

Here we do not depend on the posterior distribution of features to implement the prediction. To explain the reason we could rewrite the formula (2) as follows:

$$ML\left(t\right)\;=\;\log\;P\left(t|c^{+}\right)\;-\;\log\;P\left(t|c^{-}\right)\;=\;\log\;P\left(t,c^{+}\right)\;-\;\log\;P\left(t,c^{-}\right)\;+\;\log\;\frac{P\left(c^{+}\right)}{P\left(c^{-}\right)}$$

And the posterior distribution is:

$$\text{Posterior}\left(t\right)\;=\;\log\;P\left(c^{+}|t\right)\;-\;\log\;P\left(c^{-}|t\right)\;=\;\log\;P\left(t,c^{+}\right)\;-\;\log\;P\left(t,c^{-}\right)$$

In the *ML* schema, the relevant/irrelevant document rate $\frac{P\left(c^{+}\right)}{P\left(c^{-}\right)}$ has been taken into consideration as a compensate factor. But in the posterior probability schema, the impact of relevant/irrelevant document rate is eliminated, according to the independent and identical distribution hypothesis, while it is not tenable in our situation (since the different relevant/irrelevant rate between training and testing set).

The essence of TF*ML schema is to fully utilize the category relevant information from the annotated samples, which cannot be inferred from the TF*IDF schema. Experiment results (see Table 2) demonstrate that *ML* steadily improves the discriminative capability of features.

**Table 2:** TF*ML Feature Value Schema. The *Precision/Recall/F-Score* demonstrate classification capability of the model, and *AUC* (area under receiving operator characteristic curve) is to evaluate ranking capability of the model.

***Feature value***	***Precision***	***Recall***	***F-Score***	***AUC***
TF*IDF	0.7015	0.8213	0.7567	0.8036
TF*ML	0.7014	**0.8773**	0.7796	0.8231

(Performance under unigram feature)

### String feature

In many text classification applications, it is appealing to take every document as a string of characters rather than a bag of words [12]. Especially in bioscience, the tokenizing and stemming procedure would incur undesired loss of the informative attributions, since many of the semantically related biomedical terms that share the same stem or morpheme are often not reducible to the same stems [6]. Therefore, we propose to directly utilize the length-fixed strings as feature to exploit most of the informative segments.

To the best of our knowledge, no one explicitly takes length-fixed strings as feature because of the explosion and sparse of feature space. However, the statistical analysis based on formula (1) demonstrates that distributional divergence between the training and testing set becomes much smaller under the length-fixed string feature (see Table 3). So we turn to take the fixed-length strings as feature: the length-fixed strings are extracted from the whole sequential text without considering the sentence boundaries and strictly consist of 26 lowercase English letters (all the letters are converted to the lowercase first), 10 numbers (0-9) and a white space. *Chi-Square* statistics [13] is employed to select out the significant features and TF*IDF is computed to build the feature vector (we substitute TF*ML for TF*IDF for further improvement).

**Table 3:** KL Divergence on Training, Cross Validation and Testing Set

***String Feature(p=7)***	***Relevant Probability***	***Irrelevant Probability***
*Training Set Vs Cross Validation Set*	0.0029	0.0163
*Training Set Vs Testing Set*	0.0357	**0.1887**

(Top 50 features according to *Chi-Square* statistics)

Table 4 lists the top 10 distinct features from the selected unigram features and string features respectively. It is apparent that the length-fixed string feature has at least the following potential advantages. First, inter-word features (e.g. phrasal effects) can be exploited automatically. The segmentation process spans the boundary of adjacent words, which could exploit information from the adjacent words. Second, intra-word features (e.g. morphological variants) could be captured. For example, string “interac” would occur in the word “interact” and “interaction”, both of which are important indicator of PPI relations. Third, the special meaning of length-fixed string feature in bio-literatures is that it exploits the slight but informative commonality from the structure of the words. For example, different terminologies in bio-literatures often share the same conflation (e.g. ‘phosph’ indicates the protein phosphorylation) and most of the suffix is informative (e.g. ‘ase’ is a common suffix to proteins that function as enzyme). The specific information is not recoverable when the general tokenizing and stemming procedure is applied.

**Table 4:** Top 10 Unigram Features and String Features ‘_’ means a white space

***Unigram Feature***	***String Feature***
interaction	interac
bind	nteract
interact	_intera
domain	teracti
proteome	eractio
proteomic	proteom
complex	raction
protein	_domain
yeast	binding
kinase	_proteo

(According to *Chi-Square* statistics)

### Named entities and semantic template features

Both of the above proposed methods are domain independent, which are endowed with well generalization capacity and are not necessarily limited to the bioscience domain. But introducing domain dependent features could greatly filter out the false positive samples and further improve the performance [14]. In biological literatures, named entities (words and phrases belonging to certain predefined classes, e.g. protein and gene), such as CDC42 (protein), and semantic templates (co-occurrences of a pre-specified type of relationship between entities of given types), such as “ProteinA interact with ProteinB”, are the most meaningful concepts in PPI documents and well conserve the syntactic and semantic structures in describing the protein interactions. So we introduce the named entities and semantic templates as feature to exploit the domain dependent information.

With the help of *ABNER *[15], a named entity recognition tool, 5 types of named entities in a given document could be identified: protein, DNA, RNA, cell types and cell line. Since the recognized entity space is large and sparse, we only utilize their types as feature to decrease the dimension of feature space without losing the universality.

After recognizing the named entities, semantic templates are ready to be extracted from the documents. We propose a novel template extraction algorithm named *KeyBT*, i.e. *Key*word *B*ased *T*emplate extraction algorithm, to extract the semantic templates describing the interaction patterns among all of the recognized entities.

Compared to the traditional local alignment algorithm, *KeyBT* operates differently: first locate statistical significant words as seeds, and expand the seeds in the contextual environment iteratively, finally preserve the most “powerful” templates as the result.

The flow chart of the *KeyBT* algorithm is as follows:

1) Locate the occurrences of predefined candidate keywords in each sentence; discard the sentences without any keywords; get the initial candidate sentence set S_0_;

2) Locate each entity type in S_0_; discard the sentences without any entities; get the initial candidate template set T_0_;

3) Iteratively normalize each template in T_0_: removing the redundant templates by syntax parsing; get the raw templates set T_1_;

4) Evaluating the templates in T_1_, filter out the templates of low quality, get the final template set T_f_.

*KeyBT* not only depends on *Chi-Square* statistics to select the most distinct keywords but also utilizes *ML* to determine the category relevance of the keywords, because *Chi-Square* does not distinguish the association between features and different categories: a few high quality features of irrelevant category might be overwhelmed in the large amount of features of relevant category. *Chi-Square* is employed to select a raw candidate keyword list (with low threshold), and then top 50 features from both categories are preserved according to *ML* respectively.

We use the following formula [16] to evaluate the relevance of templates based on the balance between generality and specificity of the templates.

$$S\left(t\right)\;=\;\left(\beta \;+\;\log_{2}\;\frac{t.\mathrm{pos}\;+\;0.5}{t.\mathrm{neg}\;+\;0.5}\right)\;\times \;\ln\left(t.\mathrm{pos}\;+\;t.\mathrm{neg}\;+\;1\right)$$

where *t.pos* and *t.neg* are the positive/negative matching count of template *t* in the training set, and β is the parameter tuning the positive/negative matching rate.

When we get the final templates set T_f_, we do not simply depend on the positive/negative matching rate of each template to make the prediction. Instead, we use them to build feature vectors and train a classifier.

Top 5 *KeyBT*-extracted templates are illustrated in Table 5.

**Table 5:** *KeyBT*-extracted Templates. <PTN>, <DNA>, <CEL> mean protein, DNA and cell-line, E* means any words occurrence

***KeyBT Templates***

<PTN> E* <DNA> E* association E* <PTN>
<PTN> E* bind E* <DNA>
<PTN> E* interact E* <PTN>
<PTN> E* colocalize E* <CEL>
<PTN> E* contact E* <DNA> E* <PTN>

Compared with the local alignment algorithm that depends on the post evaluation to remove meaningless and noisy templates, the potential advantages of *KeyBT* algorithm are as follows: 1) *KeyBT* utilizes the statistical characteristic of the candidate keywords to largely remove noise before extraction; 2) *KeyBT* templates need not to fix the entities' type beforehand, so that it could catch the distribution of templates in both categories to discriminate both of the relevant and irrelevant categories; 3) the heuristic rules applied on the relation of named entities and candidate words (such as their sequence, the average template length and type of distinct entities) would guarantee the biological meaning of the extracted templates.

### Feature integration

Experiment results of the overlap among the misclassified samples by different features show that there is great complement among different features: in many cases, the false prediction caused by one feature would be treated correctly by another one. And a single type of feature is easy to lead the classifier over-fitting on the data distribution (see Table 1 and Figure 1). Thus, the integration among different features would be beneficial. In this sense, we propose two kinds of integration from different levels: feature-level and classifier-level to integrate all of above proposed features.

We perform the feature-level integration in a typical way: normalizing each part of features and unifying them into a new feature vector. We do the normalization as follows [17]:

$$\text{norm}\_\text{value}\;=\;\frac{\text{unnorm}\_\text{value }-\text{min}\_\text{value }}{\text{max}\_\text{value}-\text{min}\_\text{value}}$$

where *max_value* and *min_value* are the maximum and minimum values that are actually seen in the input feature set.

But there is an obvious defection in the above method: some lower dimensional features might be overwhelmed by the higher dimensional features (e.g. named entity feature has only 5 dimensions while length-fixed string feature has more than 10 thousand dimensions). Based on this consideration, we turn to perform the integration on the classifier level and propose two different ways to implement the integration. The first one is to integrate the output of each classifier: after training classifiers on different types of features respectively, we normalize and unify the output of each classifier into feature vectors and train a classifier. The other one is *Adaboost*[18], a general classifier integration method, which has two major advantages: firstly, *Adaboost* tunes the weight of each classifier according to its performance in each kind of training samples, which could fully utilize the discriminative capability of features; secondly, soft margin of *Adaboost* avoids the risk of over-fitting in the training process. These approaches well overcome the defection mentioned above.

## Results and discussion

The benchmark corpus is provided by BioCreAtIvE 2006. The training set contains 3536 relevant documents (title and abstract) and 1959 irrelevant. The testing set contains 750 documents, 375 of which are labelled as relevant. All of the proposed features and integration methods are implemented on the linear-kernel SVM.

### Probabilistic schema

In Table 2, TF*ML schema improves recall performance by 6.9% without losing precision compared to the traditional TF*IDF schema. The improvement validates the effectivity of exploiting the category relevance information of features and testifies *ML* to be a more effective and general feature value schema in general text classification applications.

### String feature

In our experiment, the best performance is achieved when the string length *p* is set to 7. In Table 6, the length-fixed string feature (*p*=7) gains encouraging recall improvement by more than 12.0% compared to unigram and bigram feature. But the precision has dropped about 7.2% as the expense, which can be further compensated by employing TF*ML as feature value. The practical efficiency confirms our statistical analysis of the distribution of features and gives us insight in the selection of lower level features.

**Table 6:** Length-fixed String Feature (TF*IDF)

***Feature***	***Precision***	***Recall***	***F-Score***	***AUC***
*Unigram + Bigram*	0.7015	0.8213	0.7567	0.8036
*String (p=7)*	0.6497	**0.9200**	0.7615	0.8245

(Performance under TF*IDF schema)

### Named entities and semantic template features

In Table 7, only depending on a simple criterion that if a document contains at least one protein entity, the document should be judged relevant otherwise irrelevant, we could achieve a very high recall (0.96) with an acceptable precision (0.58). Our proposed template extraction algorithm *KeyBT* well captures the complex association between the keywords and named entities and achieves promising performance in term of precision (by 11.8%) and a better improvement comparing to our former approach *ONBIRES* templates [19], which is based on local alignment algorithm.

**Table 7:** Named Entity and Semantic Template Feature

***Feature***	***Precision***	***Recall***	***F-Score***	***AUC***
*Unigram + Bigram* (TF*IDF)	0.7015	0.8213	0.7567	0.8036
*Protein Entity occurrence*	0.5815	**0.9600**	0.7243	0.7570
*ONBIRES template*	0.7647	0.7973	0.7806	0.8156
*KeyBT template*	**0.7841**	0.7653	0.7746	**0.8239**

### Feature integration

In Table 8, feature-level integration contributes the improvement in terms of *F-score* by 5.2% and *AUC* by 5.9%; in table 9, integration based on the output of classifier achieves better improvement in terms of *F-score* by 5.3% and *AUC* by 6.3%. The best performance is reached by *AdaBoost*: in *F-score* by 11.5% and in *AUC* by 8.8%. Advantage of the feature integration is obvious: different types of features are independently selected from the corpus, which focus on the different aspects of feature space and reinforce each other. From the result, it is apparent that different integration methods well leverage the capability of different types of features and achieve promising improvement.

**Table 8:** Feature-level Integration

***Feature***	***Precision***	***Recall***	***F-Score***	***AUC***
*String*	0.7044	0.8960	0.7887	0.8416
*String + Entity*	0.7360	0.8773	0.8004	0.8479
*String + Template*	0.7416	0.8880	0.8082	0.8372
*String + Entity + Template*	0.7584	0.8373	0.7959	**0.8507**

(Normalize each part of the features and unify them into new feature vectors)

**Table 9:** Classifier-level Integration. Integration on length-fixed string feature, entity feature and template feature

***Feature***	***Precision***	***Recall***	***F-Score***	***AUC***
*Unigram + Bigram*	0.7015	0.8213	0.7567	0.8036
*Output based Integration*	0.7248	0.8853	**0.7971**	**0.8539**
AdaBoost	0.7995	0.8933	**0.8438**	**0.8746**

(Normalize the output of each classifier and unify them into new feature vectors)

### Statistical significance test

Since the size of the evaluation corpus is not large enough, it is necessary to perform the statistical significance test to validate the reliability of our proposed features and integration methods. Here we employ *s-test* to evaluate the performance of systems on the pooled decisions on the individual documents/category pairs [20].

In Table 10, we can find that the proposed feature value schema TF*ML, length-fixed string feature and semantic template feature are much better than their counterpart (*p value* lower then 0.05), and two different level of feature integrations significantly improve the classification performance (*p value* lower then 0.005).

**Table 10:** Statistical Significance Test (*s-test*). The null hypothesis is that the performance of two methods is the same; the alternative hypothesis is that the former is better than the latter.

	***String Vs. Unigram+Bigram***	TF*ML Vs. TF*IDF	***KeyBT Template Vs. Unigram+Bigram***
***p value***	0.015	0.012	0.0188

	***Feature Level Integration Vs. Unigram+Bigram***		***Classifier Level Integration Vs. Unigram+Bigram***

***p value***	0.0026		0.0010

### Comparison with the state of arts

In Table 11, the mean, standard deviation and best performance from BioCreAtIvE 2006 are selected from 51 runs of 19 teams. Under our feature selection and integration procedure, the performance outperforms the previous best results (F-score improved by 8.2% and AUC improved by 2.2%).

**Table 11:** Mean, Standard Deviation and Best Performance from BioCreAtIvE 2006 Vs Our Final Performance. The best performance from BioCreAtIvE 2006 is selected from 51 runs of 19 teams respectively.

		***Precision***	***Recall***	***F-Score***	***AUC***
	*Mean*	0.6642	0.7636	0.6868	0.7351
*BioCreAtIvE 2006*	*Standard Deviation*	0.0810	0.1926	0.1035	0.0741
	*Best Reported*	-	-	0.7800	0.8554
*Final Performance*	-	0.7995	0.8933	**0.8438**	**0.8746**

## Conclusions

The experiment results clearly demonstrate that the lower level features are endowed with better generalization capability, but hampered by lower accuracy; higher level features contain rich domain dependent information, with better specificity but poor universality. Integration of different level of features would benefit from the different aspects of the feature space, which would reinforce the domain dependent classification and overcome the bias on the data distribution.

Main contributions of this paper are as follows:

(1) Propose novel domain independent feature value schema TF*ML and length-fixed string feature;

(2) Introduce domain dependent features (e.g. named entities, semantic templates) into the biological literature classification, and propose a novel template extraction algorithm *KeyBT*;

(3) Investigate the feature-level and classifier-level integration methods to incorporate the information from different levels and perspectives.

Now, the proposed methods are being integrated into our online service *ONBIRES *[21] as a pre-processing module. In the next step, we will be engaged in the aspect of incremental learning to make our approaches portable to different datasets.

## Competing interests

The authors declare that they have no competing interests.

## Authors' contributions

HW carried out the main work of the paper, proposed the methods and drafted the manuscript. MH gave directions in the whole process and revised the draft. DS participated in the design and implementation of the experiments. XZ supervised the whole work, gave a number of valuable suggestions and helped to revise the manuscript. All authors have read and approved the final manuscript.
